# ASO Author Reflections: A Simplified Classification System for In-Transit Melanoma Metastases

**DOI:** 10.1245/s10434-025-18657-z

**Published:** 2025-11-04

**Authors:** Sofia Breeze, Clare Peterson, Marc Moncrieff

**Affiliations:** 1 Norwich Medical School, University of East Anglia, Norwich, UK; 2 Department of Plastic and Reconstructive Surgery, Norfolk and Norwich University Hospital NHS Trust, Norwich, UK

## Past

In-transit metastases are a challenging manifestation of cutaneous melanoma and are frequently encountered in modern practice.^1^ Unlike nodal or distant relapse, in-transit disease usually behaves as a chronic relapsing and evolving condition. Patients may experience a gradual increase in disease burden over time yet remain within the same AJCC stage.^2^ This disconnect between disease biology and staging creates uncertainty about prognosis and contributes to variation in clinical management. Decisions about whether to continue loco-regional control strategies or escalate to systemic therapy are often based on subjective interpretation and institutional bias, rather than objective criteria. Despite therapeutic advances, there remains no dedicated framework to stratify risk or inform treatment sequencing in patients with in-transit metastases.

## Present

Our current study of 142 patients,^3^ treated within a quaternary-referral melanoma service, identified key clinical features that reflect aggressive in-transit disease behaviour. Patients with more than two lesions and/or those larger than 30 mm at initial presentation, or a short interval between treatment of the primary melanoma and development of in-transit disease (18 months or less) experienced significantly worse melanoma-specific survival. Synchronous nodal disease also correlated with poor distant metastasis-free survival. These findings suggest that not all in-transit disease is biologically equivalent, nor is the patient’s response to the disease and its subsequent treatment, and that early disease dynamics provide valuable prognostic information. By defining clinically relevant thresholds, our work offers a step towards classifying patients according to disease risk rather than anatomical pattern alone.

## Future

We are hopeful that these results provide a foundation for the development of a simplified, clinically meaningful classification system for in-transit melanoma. Validation in external cohorts is now required, alongside further exploration of molecular and immune biomarkers to refine prognostic accuracy.^4^ Ultimately, a dedicated risk stratification framework could support personalised treatment strategies, consistency in reporting outcomes and rational stratification in future clinical trials.

## References

[1] Read RL, Haydu L, Saw RPM, et al. In-transit melanoma metastases: incidence, prognosis, and the role of lymphadenectomy. *Ann Surg Oncol*. 2015;22(2):475–81. 10.1245/s10434-015-4416-4. 25256128 10.1245/s10434-014-4100-0

[2] Gershenwald JE, Scolyer RA, Hess KR, et al. Melanoma staging: evidence-based changes in the American Joint Committee on Cancer Eighth Edition Cancer Staging Manual. *CA Cancer J Clin*. 2017;67(6):472–92. 10.3322/caac.21409. 29028110 10.3322/caac.21409 PMC5978683

[3] Breeze S, Peterson CM, Garioch JM, et al. A simplified classification system for in-transit melanoma metastases. *Ann Surg Oncol*. 2025. 10.1245/s10434-025-18542-9. 41214260 10.1245/s10434-025-18542-9 PMC12901162

[4] Haywood S, Garioch J, Ramaiya A, Moncrieff M. Quantitative and spatial analysis of CD8+/PD-1 tumor-infiltrating lymphocytes as a predictive biomarker for clinical response of melanoma in-transit metastases to topical immunotherapy. *Ann Surg Oncol*. 2021;28:1029–38. 10.1245/s10434-020-08713-1. 32542563 10.1245/s10434-020-08713-1 PMC7801318

